# Improving Airways Patency and Ventilation Through Optimal Positive Pressure Identified by Noninvasive Mechanical Ventilation Titration in Mounier-Kuhn Syndrome: Protocol for an Interventional, Open-Label, Single-Arm Clinical Trial

**DOI:** 10.2196/14786

**Published:** 2020-08-14

**Authors:** Evelise Lima, Maria Aparecida Miyuki Nakamura, Pedro Rodrigues Genta, Ascedio José Rodrigues, Rodrigo Abensur Athanazio, Samia Rached, Eduardo Leite Vieira Costa, Rafael Stelmach

**Affiliations:** 1 Pulmonary Division-Heart Institute (InCor) Hospital das Clínicas da Faculdade de Medicina de São Paulo São Paulo Brazil

**Keywords:** Mounier-Kuhn syndrome, trancheobronchomegaly, tracheobronchomalacia, positive pressure, bronchoscopy

## Abstract

**Background:**

Mounier-Kuhn syndrome or congenital tracheobronchomegaly is a rare disease characterized by dilation of the trachea and the main bronchi within the thoracic cavity. The predominant signs and symptoms of the disease include coughing, purulent and abundant expectoration, dyspnea, snoring, wheezing, and recurrent respiratory infection. Symptoms of the disease in some patients are believed to be pathological manifestations arising due to resident tracheobronchomalacia. Although treatment options used for the management of this disease include inhaled bronchodilators, corticosteroids, and hypertonic solution, there is no consensus on the treatment. The use of continuous positive airway pressure (CPAP) has been reported as a potential therapeutic option for tracheobronchomalacia, but no prospective studies have demonstrated its efficacy in this condition.

**Objective:**

The purpose of this is to identify the presence of tracheobronchomalacia and an optimal CPAP pressure that reduces the tracheobronchial collapse in patients with Mounier-Kuhn syndrome and to analyze the repercussion in pulmonary ventilation. In parallel, we aim to evaluate the prevalence of obstructive sleep apnea/hypopnea syndrome.

**Methods:**

This interventional, open-label, single-arm clinical trial will enroll patients who are diagnosed Mounier-Kuhn syndrome. Patient evaluation will be conducted in an outpatient clinic and involve 3 visits. Visit 1 will involve the collection and registration of social demographic, clinical, and functional data. Visit 2 will entail polysomnography, bronchoscopy for the evaluation of tracheobronchomalacia, titration of the optimal pressure that reduces the degree of collapse of the airway, and electrical impedance tomography. In visit 3, patients exhibiting a reduction in collapse areas will be requested to undergo chest computed tomography during inspiration and forced expiration with and without positive pressure (titrated to determine optimal CPAP pressure).

**Results:**

This protocol is a doctorate project. The project was submitted to the institutional review board on January 24, 2017, and approval was granted on February 2, 2017 (Brazilian Research database number CAAE 64001317.4.000.0068). Patient evaluations started in April 2018. Planned recruitment is based on volunteers’ availability and clinical stability, and interventions will be conducted at least once a month to finish the project at the end of 2020. A preliminary analysis of each case will be performed after each intervention, but detailed results are expected to be reported in the first quarter of 2021.

**Conclusions:**

There is no consensus on the best treatment options for managing Mounier-Kuhn syndrome. The use of positive pressure could maintain patency of the collapsed airways, functioning as a “pneumatic stent” to reduce the degree of airflow obstruction. This, in turn, could promote mobilization of thoracic secretion and improve pulmonary ventilation.

**Trial Registration:**

ClinicalTrails.gov NCT03101059; https://clinicaltrials.gov/ct2/show/NCT03101059.

**International Registered Report Identifier (IRRID):**

DERR1-10.2196/14786

## Introduction

Mounier-Kuhn syndrome (MKS) or congenital tracheobronchomegaly is a chronic and rare airway morbidity associated with recurrent respiratory infections and characterized by dilation of the trachea and the main bronchi [1]. This disease was first clinically described by Mounier-Kuhn in 1932 [2]. The estimated prevalence of this syndrome in patients with pulmonary symptoms is between 0.4% and 1.6%, and it mostly affects the male gender [1,3-5]. Diagnosis of the MKS is frequently made in the third or fourth decade of life when the symptoms are more exuberant [6]. Histological alterations seen in the disease can be used to explain structural defects such as tracheobronchomalacia (TBM), saccular diverticula between cartilages, and bulging and dilation of the trachea and bronchi [1,7].

The predominant signs and symptoms of the disease include coughing, purulent and abundant expectoration, digital clubbing, dyspnea, snoring, wheezing, and recurrent respiratory infection [1]. The disease could be associated with other comorbidities such as gastroesophageal reflux disease, chronic obstructive pulmonary disease, bronchiectasis, and obstructive sleep apnea/hypopnea syndrome (OSAHS) [1,8-12].

Some of the observed symptoms could be a consequence of TBM occurrence in some patients, which is defined by more than 50% collapse of the intrathoracic trachea and bronchi [13-16]. The main clinical consequences of TBM are obstruction to expiratory airflow; secondary air trapping; decrease in the effectiveness of cough and bronchial hygiene; and facilitation of conditions that promote microorganism colonization, leading to recurrent respiratory infection [4,13].

Despite the patient’s normal pulmonary function, the occurrence of obstructive ventilatory disorder is not uncommon [1,17-19]. Thorax computed tomography (CT) shows dilatation of the trachea and the right and left main bronchi [4,20,21]. Tracheobronchial diverticula, bronchiectasis, air trapping, and emphysema are common findings, suggesting involvement of the small airways [1,7,10,11,22-26]. Bronchoscopy is the gold standard examination for TBM diagnosis [12,14-16,26].

Since TBM is a rare and poorly studied disease, there is no consensus on a specific therapy. Therapeutic management includes inhaled bronchodilators, corticosteroids, hypertonic solution, and mucolytic agents. Some complementary tools used for disease management include noninvasive positive respiratory pressure, vaccination, respiratory physiotherapy, and pulmonary rehabilitation [1,27-29]. Continuous positive airways pressure (CPAP) support has been reported as an option for the treatment of tracheomalacia [5,15,30]. The rationale for using positive pressure in TBM is to recover the patency of the collapsed airways, thereby reducing airflow obstruction and promoting sputum clearance.

Previous experience has shown that CPAP can reduce symptoms, infections, and morbidity/mortality caused by TBM [5,15,31-33]. Additionally, it is an effective therapy in patients diagnosed with concomitant OSAHS [34-36]. This study has two main objectives. First, we aim to confirm the presence of tracheal collapse and the main bronchi of patients with MKS through respiratory endoscopy. Second, we will attempt to optimize the pressure with the CPAP approach to reduce or reverse the pathological consequences of the syndrome on the ventilatory pattern. We also will investigate the prevalence of OSAHS in this population. We hypothesize that maintenance of greater patency of airways could improve pulmonary ventilation for a longer time, reduce symptoms, and improve the quality of life of these patients.

## Methods

### Overview

This is an interventional, open-label, single-arm clinical trial to be conducted on patients diagnosed with MKS, who are followed up in the outpatient clinic from a tertiary university hospital. Eligible patients diagnosed with MKS who are followed up and agree to participate in the study will be requested to sign an informed consent form after verbal explanation and clarification of the study are provided by the investigator.

The following patients will be excluded from the study: those who are contraindicated for bronchoscopy (with thrombocytopenia, incorrigible coagulopathies, refractory hypoxemia, recent acute myocardial infarction, unstable angina, acute cardiac arrhythmias, and refractory bronchospasm), pregnant women under the age of 18 years, and those suspected of having or diagnosed with mycobacteriosis or other related risk factors by the clinical investigator. The study is approved by the institution's ethical review board and funded by the Division of Pulmonology Obstruction Group.

### Study Design

The study will be conducted in three steps including three patient visits. During visit 1 (day 1), sociodemographic information, modified Medical Research Council dyspnea questionnaire measures [37-39], the Saint Georges Respiratory questionnaire measures [40,41] for health-related quality of life, and most recent spirometry values will be recorded. In addition, all eligible patients will receive 40 mg/day oral prednisone for 7 days before endoscopy, to reduce pulmonary secretion and possible airways hyperreactivity. If the patient presents signs or symptoms that characterize an acute pulmonary infection, the inclusion will be postponed, and specific treatment will be prescribed to reschedule the inclusion.

During visit 2 (day 7 ± 3 days from visit 1), polysomnography and respiratory endoscopy will be performed. Full-night polysomnography will be performed in accordance with the recommendations of the American Academy of Sleep Medicine [42,43].

After polysomnography, a strap of 32 self-adhesive electrodes strap will be attached at the level of fourth intercostal space around the chest, and patients’ electrical impedance tomography data will be recorded concomitantly with bronchoscopy. Bronchoscopy will be performed in two stages: (1) sedation, analgesia, and evaluation of the presence of TBM followed by bronchial hygiene and (2) optimal pressure titrations to decrease airway collapse and EIT performance. The procedure sequence will be conducted in two stages, as described below.

#### Stage 1

The patient will be placed in a dorsal position for cardiac monitoring, noninvasive blood pressure measurement, and pulse oximetry. The nasal fossae will be lubricated with 2% topical lidocaine gel, followed by the introduction of nasopharyngeal catheter number 08 to provide supplemental oxygen. Fentanyl (0.5-2 μg/kg) and midazolam (0.03-0.05 μg/kg) will be administered intravenously to achieve light sedation. Propofol bolus (20-40 mg/dose) may be used to achieve the desired sedation. Next, the custom nasal mask of CPAP will be attached to the patient’s face, allowing the passage of the video-bronchoscope (Standard Q180 Olympus) with an external diameter of 4.9 mm and a working channel of 2.0 mm. Topical lidocaine at a dose of 1% without a vasoconstrictor (maximum dose of 7 mg/kg) will be instilled in the larynx and tracheobronchial tree. This will be followed by secretion aspiration. Subsequently, the presence of fixed or dynamic tracheal and bronchial collapse during the respiratory cycle will be analyzed and recorded. Direct observational assessment will be defined at the site with the greatest tracheal collapse and main bronchi during inspiratory and expiratory normal ventilation. Patients who do not present significant airway collapse will be excluded from the next step.

#### Stage 2

**Figure 1:**
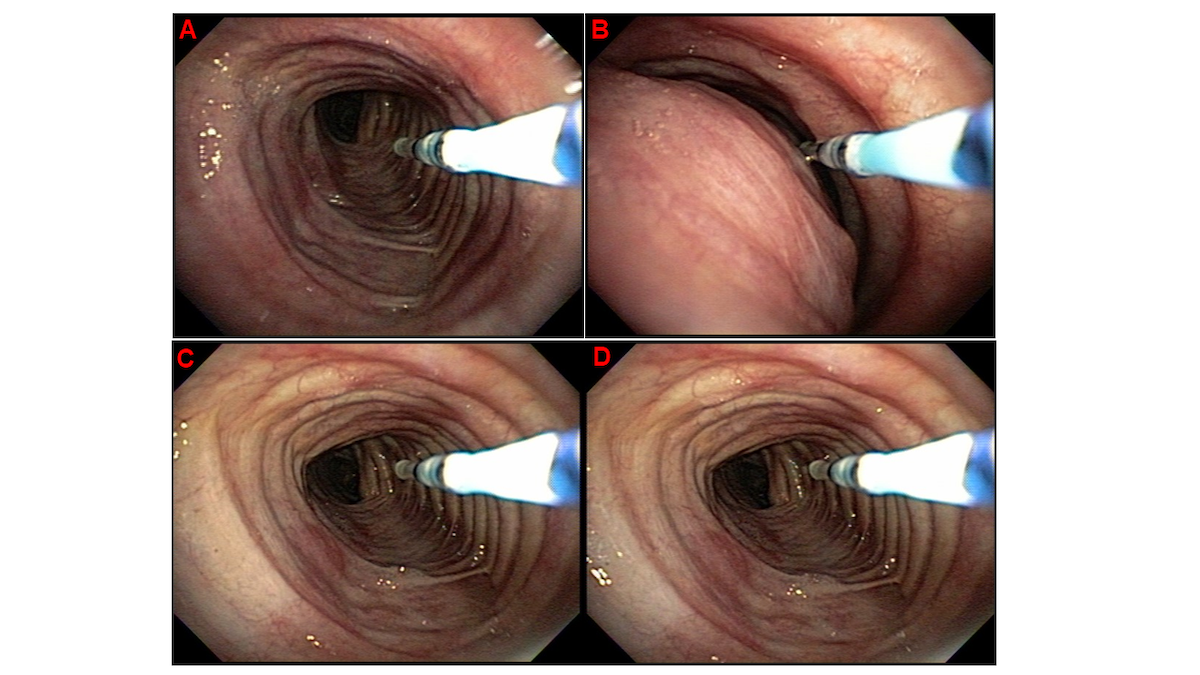
Bronchoscopic view of the trachea and the catheter (blue). Trachea during 0 cm H2O pressure during inspiration (A) and during expiration (B). Trachea in 18 cm H_2_O pressure during inspiration (C) and expiration (D)

To evaluate the reduction of airway collapse, the bronchoscope will be fixed with the aid of a customized catheter in the tracheal region at least 2 cm before the area of the greatest collapse, previously defined by three bronchoscopists present in the examination room. The CPAP titration will start with a pressure of 0 cm H2O and be gradually increased in steps by 2 cm H2O every 10 complete respiratory cycles until a pressure of 18 cm H2O is reached (Figure 1).

 Through observational analysis and after a complete consensus among the bronchoscopists is reached, the minor pressure capable of reducing the degree of collapse in the trachea will be defined (P1). This procedure will be repeated in the main bronchus presenting major collapse. The entire procedure will be documented through video recording, and the images will be analyzed with software assistance (Image Processing Toolbox, Matlab). Measures in the airway variation area will be analyzed by comparing the area of collapse at 0 cm H2O pressure with the corresponding measurements at different applied pressures titrated during the procedure. This analysis will allow us to find the pressure that reduces the degree of airway collapse (P2) [42,43]. EIT will be used for impact analysis of different pressures on pulmonary ventilation (distal airways), a functional imaging method that uses low-intensity electric currents. EIT can dynamically evaluate regional pulmonary ventilation by analyzing impedance variations and minimum impedance in a given thoracic segment by reproducing them in two-dimensional images. It is a noninvasive method that does not use ionizing radiation [44-47].

The EIT data will be acquired using ENLIGHT (Timpel), which produces 50 images per second, sampled in real time [44-47]. Distribution of ventilation will be analyzed at each CPAP level during the positive and expiratory pressure (PEEP) titration maneuver by dividing the EIT image into 4 quadrants (regions of interest, ROI), 2 of which will be gravity dependent (lower lobes) and 2 will be gravity independent (upper lobes). In patients who present tracheomalacia during bronchoscopy, a chest CT will be performed at visit 3 during inspiration and forced expiration in the presence or absence of CPAP. The ideal pressure for this will be obtained during bronchoscopy with an aim to evaluate and document the reduction of tracheal collapse. The images will be obtained by multislice tomography. No intravenous contrast will be administrated.

### Statistical Analysis

Because MKS is a rare disease, there will be no sample size calculation. It will be a convenience sample including all eligible patients from Mounier-Kuhn reference centers in Brazil. As of July 2020, we are following at least 15 patients with MKS.

We will perform five main types of descriptive analysis on the data: (1) sociodemographic data of all MKS populations included in the study; (2) comparative analysis of the clinical-functional data of patients with and without collapse; (3) prevalence of sleep apnea/hypopnea syndrome; (4) repeated measurement techniques (bidirectional analysis of variance and Tukey or Sidak post hoc test when there is a difference) correlating the pressure that reverses the collapse (P1 and P2), to analyze the difference between airway collapse that occurs with and without PEEP; and (5) comparisons in the distribution of EIT ventilation with and without PEEP. Statistical analysis will be performed with statistical packages Sigma Stat V3 or SPSS V22, and an α level ≤.05 will be considered significant for all tests.

## Results

The project was submitted to the institutional review board on January 24, 2017, and approved on February 2, 2017 (Brazilian Research database number CAAE 64001317.4.000.0068). The project was registered in ClinicalTrails.gov (NCT03101059) on March 23, 2017. This protocol is a doctorate project. Patient evaluations started in April 2018. Planned recruitment is based on volunteers’ availability and clinical stability, and the interventions will be conducted at least once a month and completed at the end of 2020. A preliminary analysis of each case will be performed after each intervention, but detailed results are expected to be closed in the first quarter of 2021. We plan to publish 3 papers on this project. The proposed schedule for publication of the papers is as follows: the intervention protocol in 2019-2020; literature review about the concept of tracheobronchomalacia and airway collapse according to diverse uses ways to measure them and their definition in 2020; and results of the presence of tracheal collapse and the main bronchi of patients with MKS, through respiratory endoscopy and optimum positive pressure with CPAP that reduces or reverse it, in 2021.

## Discussion

Thus far, the main finding of this study is the identification of the progressive increase in the degree of pressure that can reverse the collapse of the trachea and bronchi and stabilize the airway.

MKS or congenital TBM is a rare and poorly studied disease. It has a wide spectrum of signs and symptoms and can have a great impact on the quality of life. Dyspnea, accumulation of secretions in the airways, bronchiectasis, and recurrent respiratory infections increase the morbimortality of the disease.

There is no consensus on the best treatment. In general, measures extrapolated from other pulmonary pathologies, such as those employed in chronic obstructive pulmonary disease and bronchiectasis, are used for therapeutic management. Among the specific treatments currently available, we want to highlight the endotracheal and endobronchial prosthesis and tracheobroncoplasty surgery. However, both approaches have limited results and are associated with frequent complications.

There is a rationale for the use of positive pressure. The use of positive pressure could maintain the patency of the collapsed airways, thereby providing a “pneumatic stent,” which in turn could reduce airflow obstruction and promote mobilization of secretions within the airways. However, no study protocol has been performed to test this hypothesis.

The goal of this study is to assess if positive pressure with CPAP can be applied to reduce the extent of airway collapse and improve ventilation in MKS patients with tracheobronchomalacia. At the same time, we will evaluate the prevalence of OSAHS, a related and frequently occurring comorbidity that contributes to the worsening quality of life of patients with MKS.

## Abbreviations

CPAP: continuous positive airway pressure
CT: computed tomography
EIT: electrical impedance tomography
MKS: Mounier-Kuhn syndrome
OSAHS: obstructive sleep apnea-hypopnea syndrome
TBM: tracheobronchomalacia
